# Quantification of Circadian Rhythms in Single Cells

**DOI:** 10.1371/journal.pcbi.1000580

**Published:** 2009-11-26

**Authors:** Pål O. Westermark, David K. Welsh, Hitoshi Okamura, Hanspeter Herzel

**Affiliations:** 1 Institute for Theoretical Biology, Humboldt University, Berlin, Germany; 2 Department of Psychiatry, University of California San Diego, La Jolla, California, United States of America; 3 Veterans Affairs San Diego Healthcare System, San Diego, California, United States of America; 4 Department of Systems Biology, Graduate School of Pharmacological Science, Kyoto University, Kyoto, Japan; EMBL, Germany

## Abstract

Bioluminescence techniques allow accurate monitoring of the circadian clock in single cells. We have analyzed bioluminescence data of *Per* gene expression in mouse SCN neurons and fibroblasts. From these data, we extracted parameters such as damping rate and noise intensity using two simple mathematical models, one describing a damped oscillator driven by noise, and one describing a self-sustained noisy oscillator. Both models describe the data well and enabled us to quantitatively characterize both wild-type cells and several mutants. It has been suggested that the circadian clock is self-sustained at the single cell level, but we conclude that present data are not sufficient to determine whether the circadian clock of single SCN neurons and fibroblasts is a damped or a self-sustained oscillator. We show how to settle this question, however, by testing the models' predictions of different phases and amplitudes in response to a periodic entrainment signal (zeitgeber).

## Author Summary

Earth's 24-h-rotation around its axis is mirrored in the circadian clock that resides within each of our cells, controlling expression of ∼10% of all genes. The circadian clock is constructed as a negative feedback loop, in which clock proteins inhibit their own synthesis. During the last decade, a picture has emerged in which each cell is a self-sustained circadian oscillator that runs even without synchronizing cues. Here, we investigated state-of-the-art single-cell bioluminescence recordings of clock gene expression. It turns out that these time series are very well described by low-dimensional models, enabling us to extract descriptive parameters that characterize each cell. We find that different cell types do not differ much in their dynamics. However, different mutations in core clock genes yield different dynamic characteristics. Furthermore, we could not statistically reject the idea that the cells are in fact damped oscillators driven by noise. We thus declare the question of whether the circadian clock is a damped or self-sustained oscillator still unresolved. Further, we propose a way to resolve this question by examining the frequency-dependent response of single cells to periodic stimuli. We will then be in a better position to understand how cells coordinate and synchronize their circadian rhythms.

## Introduction

The circadian rhythm of organisms ranging from cyanobacteria to humans beats at the cellular level; it is a remarkable manifestation of celestial mechanics mirrored in molecular biology. The standard view is that the mammalian circadian clock is a hierarchically organized system, governed by the suprachiasmatic nuclei (SCN, consisting of about 20,000 neurons) in the hypothalamus. The SCN neurons are coupled to each other and are entrained by light to oscillate in synchrony to the 24 h earth rotation, and in turn entrain cells and organs in the rest of the body. In studies of dissociated SCN neurons, typically most of the cells are classified as being self-sustained oscillators [1]–[4]. However, as also predicted theoretically [5],[6], dissociated individual oscillating SCN neurons can vary greatly in their precision, and many have been suggested to be damped oscillators [1],[7], especially if synaptic input or 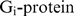 mediated signaling is compromised [8],[9]. At the SCN tissue level, mutant neurons that are arrhythmic when dissociated from each other can interact to generate a collective coordinated self-sustained rhythm [10].

Peripheral tissues contain independent clocks [11], thought to be synchronized by the SCN via neural and hormonal pathways, as well as via more indirect routes such as body temperature and feeding behavior [12]–[14]. In the last decade it became clear that the circadian rhythm of immortalized fibroblast cell lines [15] as well as peripheral tissues such as liver, lung, and muscle [11], has its origin at the single cell level. These rhythms are damped at the cell population level, but recent studies employing single-cell techniques suggested that the rhythms in peripheral tissues actually are self-sustained at the single-cell level [16]–[17]. It is not clear how the peripheral rhythms may differ from the SCN rhythm at the single-cell level.

Rapid progress has been made during the last decade in unraveling the molecular components of the clock, although the picture is not yet complete. The consensus view is that the core clockwork consists of several interlocked negative and positive feedback loops [18]. There are numerous theoretical models for how these combine into a ticking molecular clock, all of which assume a self-sustained oscillator that essentially relies on the negative feedback loop consisting of the Period (PER1, PER2) and Cryptochrome (CRY1, CRY2) proteins inhibiting their own production once translocated into the nucleus, by attenuating the action of their transcriptional activators CLOCK and BMAL1 [19]–[22]. Corresponding knockout mutants have been studied at the cellular level [10], although a quantitative characterization of these mutants is lacking.

Using two simple canonical oscillator models and time-series analysis, we have extracted general parameters, such as oscillator damping rates and biochemical noise levels, from previously published single-cell bioluminescence measurements in SCN neurons and fibroblasts [9]–[10]. Considerable progress has recently been made in the understanding of the origin of noise in gene expression and protein concentrations [23], and such noise was considered in our analysis since it is readily noticeable and quantifiable from the data at hand. We report here that the circadian clock in both wild-type single dissociated SCN neurons and in fibroblasts is well approximated as a damped (non-self-sustained) oscillator driven by biochemical noise. This model assumes that the underlying dynamics of the oscillator are those of a damped system, but that the damped oscillator via the filtering and molding of biochemical noise exhibits a circadian rhythm. An alternative minimal self-sustained (limit-cycle) oscillator model described SCN neurons as noisy small-amplitude oscillators with noise levels comparable to the oscillator amplitudes. Similar low-dimensional self-sustained models have previously been applied successfully to analysis of circadian single-cell fluorescence and bioluminescence time series [24]–[26], as well as to measurements of circadian body temperature cycles [27],[28].

We were also able to characterize 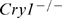, 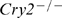, and 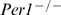 mutants, to each of which we could confer a unique quantitative signature. This paves the way for a characterization of mutants that goes beyond the mere classification as “rhythmic” or “arrhythmic”. Finally, based on a comparison with bioluminescence recordings of single neurons within cultured SCN tissue slices, we show how to use the fitted models to understand entrainment properties of single cells. Importantly, the entrainment properties differ markedly, depending on whether the noise-driven damped model or the self-sustained model is assumed, pointing to future experimental tests that would discriminate between the two concepts.

## Results

### Single cell circadian rhythms are well described by a linear damped oscillator model driven by biochemical noise

**Figure 1:**
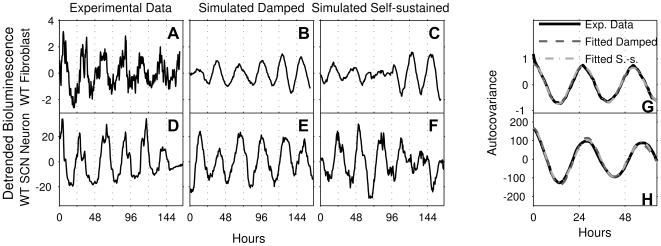
Detrended, mean-centered experimental data, simulations of damped and self-sustained models, and estimated and fitted autocovariance functions. (A and D). Representative time-series of a WT fibroblast (A) and SCN neuron (D). Raw data were detrended by subtracting a least-squares fit of a second-degree polynomial and then mean-centered. The unit for the bioluminescence is photons per minute. (B and E). Simulations of the damped model (Equation 3) with parameters extracted from the autocovariance estimations of the time-series in panels A and D, respectively. (C and F). Simulations of the self-sustained model (Equation 5) with parameters extracted from the autocovariance estimations of the time-series in panels (A) and (D), respectively. (G and H). Autocovariances were estimated from the time-series in panels (A) and (D), respectively, using an unbiased estimator (black solid curves). Fits to the estimated autocovariances of the theoretical autocovariance functions for the damped model (Equation 4) are shown as dark gray dashed curves. Fits for the self-sustained model (Equation 6) are shown as light gray dashed curves. The fitting procedure is described in Text S1.

**Figure 2:**
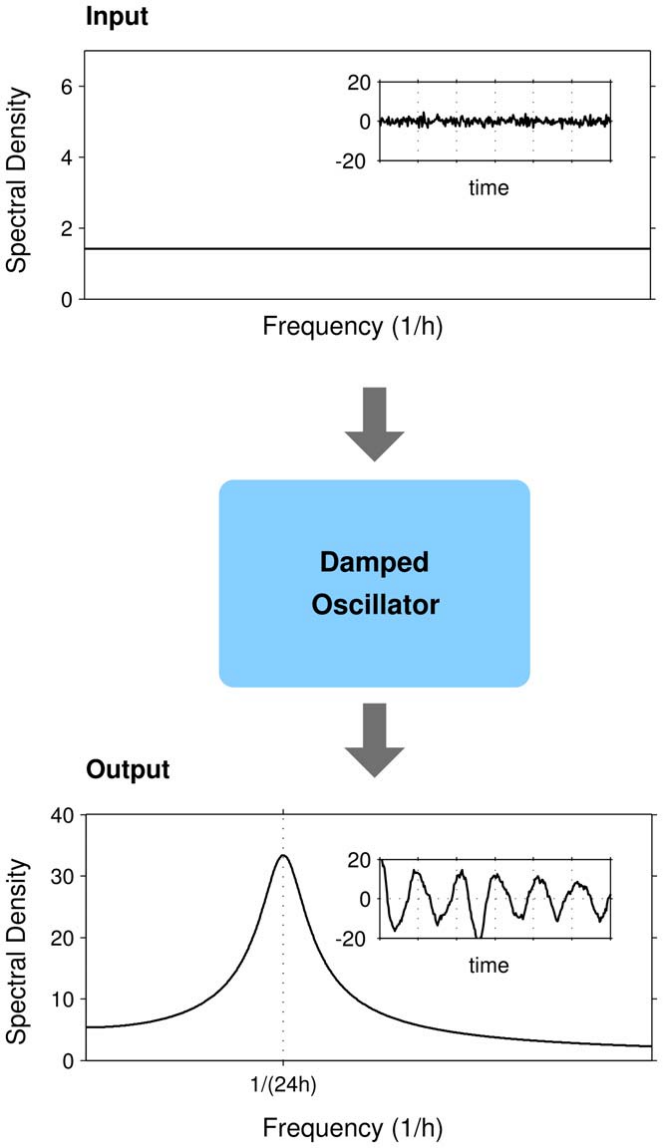
Noise can generate well-defined oscillations in a damped oscillator. White noise (upper panel) has a constant spectral density for all frequencies. It enters the damped oscillator and becomes filtered by it. The resulting amplitude spectral density of the variable 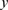, as calculated from Equation S2 in Text S1, is drawn in the lower panel. The shape of the spectral density of 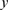 is entirely determined by the parameters of the damped oscillator, here chosen as 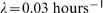 and 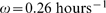 which are typical values of the fits of the damped model to the bioluminescence data. Insets show input white noise (upper panel, 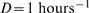), and a corresponding simulated time series for the 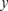 variable (lower panel, 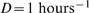, 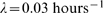, 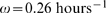).

We used raw data from a recent investigation [10], where bioluminescence imaging was employed to track the relative amount of functional mPer2-luciferase fusion protein in single dissociated mouse SCN neurons and fibroblasts, for wild-type (WT, fibroblasts: 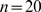, SCN neurons: 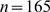) and for 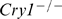 (fibroblasts: 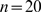, SCN neurons: 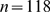), 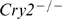 (fibroblasts: 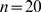, SCN neurons: 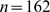), and 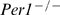 (fibroblasts: 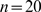, SCN neurons: 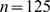) mutants. Example WT time-series (detrended and mean-centered) are shown in Figures 1A (fibroblast) and 1D (SCN neuron). From the bioluminescence time-series, we calculated empirical autocovariances and fitted these to the theoretical autocovariance formula of a linear damped oscillator with additive noise (Methods). This model is comprised of two variables that oscillate, with a given period, towards a stable equilibrium point. However, the additive noise keeps the variables from settling at the equilibrium point, continuously offsetting them, which makes the oscillator appear to be self-sustained. In effect, the biochemical noise is filtered by the damped oscillator so that the circadian frequency components are amplified, while other frequency components are suppressed. This principle is illustrated in Figure 2.

The model is specified by three parameters only: the frequency, the damping rate, and the noise intensity. A representative example of the fitting of the autocovariances is given in Figures 1G and 1H, where the experimental data autocovariances (black lines) were calculated from the time-series in Figures 1A and 1D, respectively. In general, excellent fits were obtained with the linear damped oscillator model. With the fitted parameters, the model can be used to simulate time-courses (see Methods and Text S1 for details about the model and simulations), as shown in Figures 1B (fibroblast) and 1E (SCN neuron). The simulations illustrate that the noisy damped linear oscillator model indeed produces time courses qualitatively similar to the experimental ones (Figures 1A and 1D). Note that the seemingly lower simulated noise level in Figure 1B compared to the experimental data in Figure 1A is due to the fitting procedure being able to selectively filter out and reject measurement noise (see Text S1). In order to test whether we could reject the damped oscillator model we adopted a bootstrapping approach related to the method proposed by Hall and Wilson [29]: we made 1000 simulations of the model (Methods) for each cell, using the parameters extracted from the data. For each simulated time-series, we calculated autocovariances and again fitted these to the analytical autocovariance function (Methods) as outlined above. Thus, for each cell, we have one autocovariance function calculated from experimental data, and 1000 autocovariance functions calculated from simulations. We then calculated the fraction of the 1000 simulations that produced better fits (in the least-squares sense) than the experimental data. We took this fraction as a measure of how reasonable the model is: if the simulations give better fits than experimental data in more than 95% of the cases, we consider the experimental data to be different enough from the simulated time series to reject the damped model. We could reject the damped model in this way for only 5.1% of the SCN neurons. For fibroblasts, the percentage was higher, 17%. The full results are given in Table S1.

**Figure 3:**
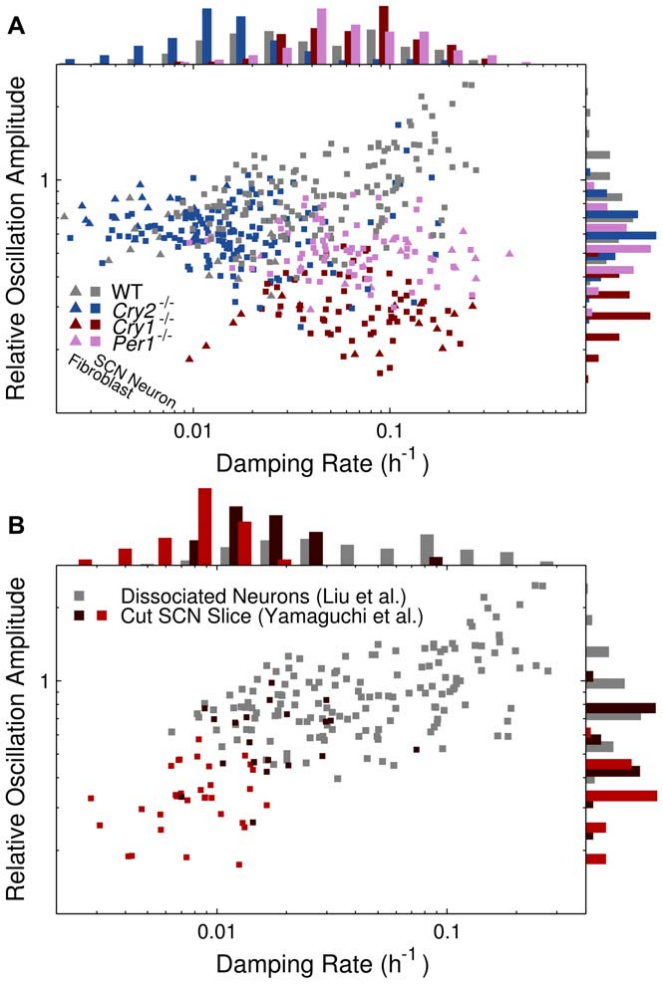
Summary of parameter estimations for the damped model. The quantitative signature of each cell according to the damped model is visualized in two dimensions. On the abscissas are the estimated damping rates 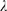; the smaller this value, the higher the fidelity of the damped oscillator. For a given value of 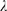, the average oscillation amplitude is proportional to the square root of the noise intensity 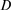, since the oscillations of the damped model are driven by noise. On the ordinates are the relative oscillation amplitudes, i.e. oscillation amplitudes divided by overall means of the respective bioluminescence signals. Relative (dimensionless) units are given, since these make the comparison between different experimental settings possible. (A). Data for cells studied by Liu et al. [10]. The cells cluster according to mutants (WT, 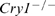, 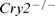, and 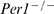), rather than type (SCN neurons or fibroblasts). It is furthermore clearly visible that 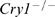 and 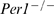 mutant cells occupy different parts of parameter space, which was not noted before. (B). The WT SCN neurons in the study of Liu et al. [10] together with the WT SCN neurons of the smaller, nonsynchronized ventral parts of two different SCN slices cut in two, from the study of Yamaguchi et al. [9] (brown and red points, respectively). These different data were recorded in different experimental settings in different laboratories. Nevertheless, these different neurons occupy the same region in parameter space, although the cells from the cut SCN slices are seen to have generally smaller amplitudes and smaller damping rates.

To summarize and visualize the data fits for all cells in the Liu et al. [10] study, the fitted damping rates are plotted against the average noise-driven relative oscillation amplitudes in Figure 3A. Relative amplitudes are the oscillation amplitude divided by the overall mean. Key results of this parameterization are: (1) We can characterize WT, 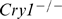, 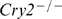, and 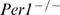 mutants, i.e. separate them in parameter space. 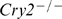 mutants were more weakly damped than WT cells and the other two null mutants. 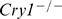 mutants have a more disrupted circadian rhythm than 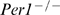 mutants, since their average relative oscillation amplitude is distinctly lower. This difference was not noted earlier [10]. (2) SCN neurons and fibroblasts cluster together, both for WT and mutant cells. Thus, given this model, we can find no significant difference between the circadian rhythms of these two cell types at the uncoupled single-cell level. Although fibroblasts generally give rise to dimmer bioluminescence due to lower PER2 expression level, cf. panels 1A and D, this peculiarity is not reflected in our analysis, since we measure amplitudes in relative units. (3) According to this model and data fit, WT cells constitute a more heterogeneous population than any of the mutants, in that the parameters of the model exhibit a larger spread. We could quantify this, cf. Table S2. Ordered according to heterogeneity, 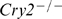 were less heterogeneous, 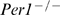 mutants even less heterogeneous, while the 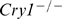 mutants were the most homogeneous. (4) The damping time of the cells, i.e. the time it would take a given perturbation to decay to half its magnitude, lies on the order of 5 to 100 hours with a median of 21 hours. As discussed below, this has implications for the synchronization and entrainment of the cells.

To probe the generalizability of the method and the results, we further analyzed hitherto unpublished bioluminescence data from an earlier study by Yamaguchi et al. [9], where transgenic mice with a luciferase reporter gene driven by the *mPer1* promoter were used. The data we analyzed come from the smaller ventral part of two mouse SCN slices each cut in two. The cells in the smaller parts were not synchronized, and we hence infer that they may have been in a state similar to the dissociated SCN neurons from the Liu et al. [10] study. Again, we calculated the autocovariance for each cell (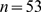), and fitted these to the theoretical autocovariance function for the linear damped oscillator (Methods). The results are summarized in Figure 3B, where we have plotted the results of these fits together with those of the earlier fits to the WT cells of the Liu et al. [10] study. It is most reasonable to consider relative amplitudes when comparing data from different studies, since relative units are insensitive to differences in expression level of reporter genes, laboratory equipment, and other systematic factors. In general, although different reporter genes were used in the two studies, the cells occupy the same region in the parameter space of our model. We also performed the statistical test outlined above for these cells; in this case, we could reject the damped model for 1.9% of the cells.

### An alternative, self-sustained oscillator model describes the single-cell circadian rhythm as noise-dominated

We sought to use the same methodology as above to evaluate the consensus picture of the circadian clock as a self-sustained oscillator at the single-cell level. This can be modeled most simply as a two-variable oscillator with a given amplitude, a given noise intensity, and a given amplitude relaxation rate to the stable oscillation (limit-cycle, Methods). Also in this case, one obtains a theoretical autocovariance formula from the model, which was fitted to the autocovariances estimated from the experimental time-series, as in the representative example in Figure 1G and H, light gray dashed line. The autocovariance formula for the self-sustained oscillator model has two degrees of freedom more than that of the linear damped oscillator (Methods), which is why we always expect a slightly better fit in the self-sustained case. Again, this model can be simulated (see Text S1), and typical simulated time-courses are shown in Figures 1C and F (fibroblast and SCN neuron, respectively), which show that the self-sustained model also generates realistic time-courses. As for the damped model, we took the bootstrapping approach described above and made 1000 simulations for each cell and tested for how many cells we could reject the self-sustained model. This we could do for 4.2% of the SCN neurons and for 18% of the fibroblasts. The full results can be found in Table S1.

**Figure 4:**
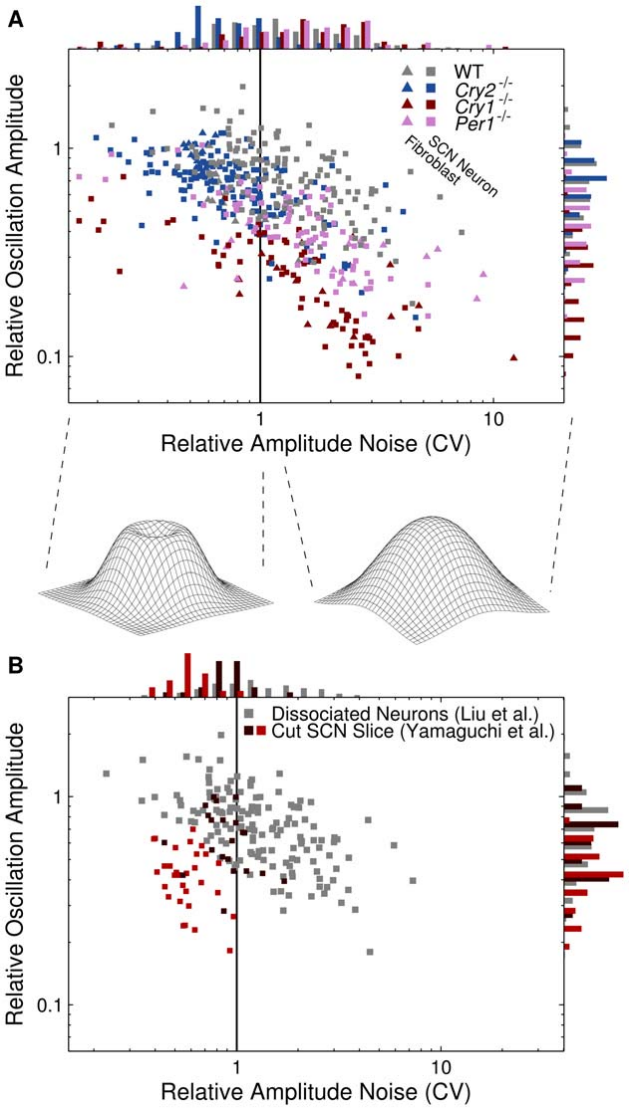
Summary of parameter estimations for the self-sustained model. Here, the quantitative signature of each cell according to the self-sustained model is given in two dimensions. On the abscissas, the amplitude CVs, i.e. the amplitude fluctuation SDs divided by the estimated amplitudes 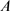 (which is a parameter of the self-sustained model, see Methods), are given. If this quantity exceeds 1 (solid vertical lines in the panels), we consider the oscillator to be noise dominated, since its stationary probability density has a single maximum (schematically drawn below panel A). If the quantity is less than 1, however, the stationary probability density forms a “crater ridge” (also schematically drawn below panel A) representing the stable oscillation (limit cycle). On the ordinates are the relative oscillation amplitudes, i.e. amplitudes 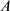 divided by overall means of the respective bioluminescence signals. (A). Here, the cells in the study of Liu et al. [10] are visualized. Again, the cells cluster according to mutants (WT, 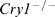, 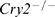, and 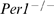), rather than type (SCN neurons or fibroblasts). Further, 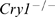 and 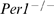 mutant cells are markedly noisier than the other genotypes, with the vast majority of cells being noise dominated oscillators. On the other hand, 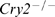 mutants are in general not noise dominated, but have stationary probability densities exhibiting oscillator crater ridges. WT cells occupy the middle ground, with 59% of the cells being noise dominated. (B). The WT SCN neurons in the study of Liu et al. [10] (grey points) together with the WT SCN neurons of the nonsynchronized SCN neurons from the study of Yamaguchi et al. [9] (brown and red points). The neurons from the two different studies occupy the same region in parameter space, although the neurons from the Yamaguchi et al. [9] study have lower relative amplitudes and are less noisy. The majority of these neurons are not noise dominated.

We summarize the results from these data fits in Figure 4. On the abscissa are the coefficients of variation (CV) for the amplitudes, which are the standard deviations (SDs) of the fluctuations in the oscillator amplitudes, divided by the amplitudes themselves. On the ordinate are the oscillator amplitudes divided by the means of the time-series, as for the fits to the damped model. Of special note is that if the CV is greater than 1, the stationary probability density (see Text S1) of the two variables of the oscillator is qualitatively the same as for a damped oscillator–it is unimodal, i.e. has one single maximum. However, if the CV is less than 1, the probability density forms a “crater ridge” around a local minimum. This “crater ridge” represents the self-sustained oscillation. The key results for the data fit to the self-sustained oscillator model are: (1) In this model, as in the damped model, the WT cells and mutants are also clearly separable. The 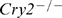 mutant cells generally have clear oscillator characteristics (CV less than 1), while the majority of the WT (58%) and other mutant cells have a CV greater than 1, meaning that the amplitude fluctuation SDs are greater than the oscillator amplitudes. Thus the stationary probability densities are unimodal just like for damped oscillators. Again, 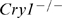 mutants generally had a weaker rhythm than 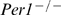 mutants. (2) As above, the fits to the self-sustained model cluster the fibroblasts together with the SCN neurons. (3) For the parametrization chosen in Figure 4, WT cells are again the most heterogeneous. Ordered according to heterogeneity, 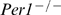 mutants were less heterogeneous, 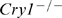 neurons were even less heterogeneous, while 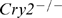 mutants were the most homogeneous (see Table S2). (4) The damping time for relaxation to the oscillation cycle is on the order of 1 to 10 hours with a median of 3.2 hours.

We repeated the same fitting procedure for the neurons of the Yamaguchi et al. [9] study. The results, overlayed by the results of the fit to the WT cells of Liu et al. [10], are shown in Figure 4B. Just as for the damped model, the cells from the two different studies occupy the same region in parameter space, although we see here that the neurons of the Yamaguchi et al. [9] study generally are less noisy; most of them have a CV less than one. Also, there is a trend towards lower relative amplitudes, which at least partially could be due to a higher background glow from non-synchronized neighboring neurons of the intact slice. Statistically, we could reject the self-sustained model (using the method outlined above) for 26% of these neurons.

### The models explain entrainment phases and predict different frequency response curves

#### Resonance

For the linear damped oscillator model, elementary theory can be used to calculate the amplitude of a given oscillator when driven by an incoming sinusoidal oscillatory signal. In the present case, this conceptually corresponds to, for example, an SCN neuron *in vivo* or in slice cultures being synchronized by other SCN neurons. The linear damped oscillator amplifies (or attenuates) the absolute amplitude of the incoming signal with a factor–a gain 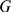
–that depends on the frequency of the signal. Plotting 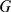 as a function of the frequency 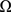 of the incoming signal results in a frequency response curve (Methods). If 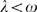, where 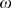 is the natural (non-entrained) oscillator frequency and 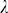 is the damping rate, this curve has a maximum at the resonance frequency 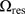, at which
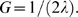
(1)


The smaller the damping rate, the higher and also sharper the resonance peak becomes. A standard characteristic of a frequency response curve is the 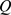 factor, which is the gain at the resonance frequency (if there is one) divided by the gain at zero frequency (static signal):
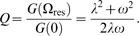
(2)


Often, it is preferable to study relative amplitudes, which are defined as the oscillation amplitude divided by the overall mean of a signal. The 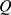 factor is in fact the gain of the relative amplitude at the resonance frequency, i.e. the relative amplitude of the entrained oscillator divided by the relative amplitude of the entrainment signal. 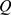 is therefore particularly suitable as an experimental measure.

Also the self-sustained model may be approximated as a linear damped oscillator, if subject to an input signal in such a way that the amplitude is significantly greater than in the non-driven case. We then consider the oscillator to be in a regime where the damping towards the non-driven self-sustained oscillation (limit cycle) can be approximated as the damping towards a stable equilibrium point. In this approximation, we treat the low-amplitude self-sustained oscillator as being *essentially damped*. This makes particular sense when the amplitude fluctuation SD is greater than the oscillator amplitude (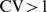). As discussed below and in Text S1, we performed simulations to test the validity of this approximation.

Since the estimated damping rates 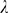 for the two different models lie in different regimes, we anticipate, according to Equations 1 and 2, the entrainment behavior of the two models to be different. This is investigated in detail below.

Additionally, this view of the oscillators allows the calculation of entrainment phases of each cell. The entrainment phase is defined as the phase difference between an entrained oscillator and an entrainment signal. This calculation requires only the frequency 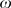 of the oscillator, its damping rate 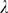 and the frequency 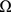 of the entrainment signal (Equation 9, Methods).

#### Estimating entrainment phases

**Figure 5:**
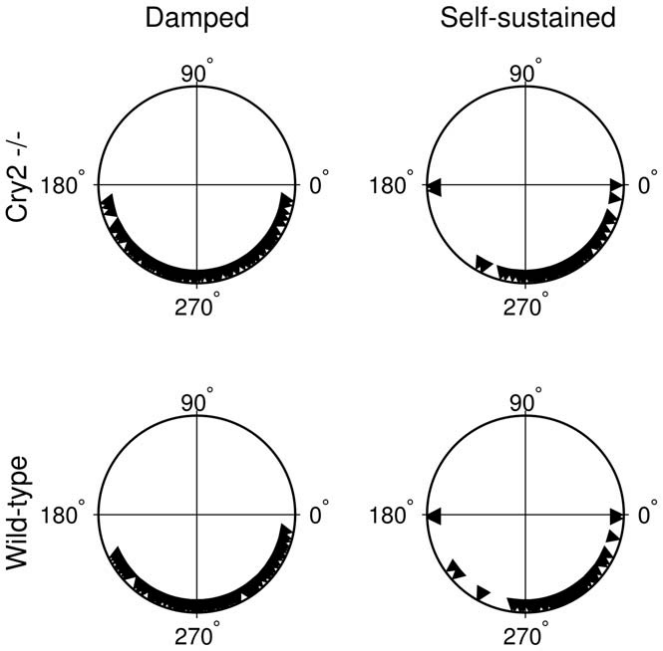
The models explain observed entrainment phases. The predicted phases of entrainment for the Liu et al. [10] WT SCN neurons (compare with Figures 2B and 3E in the article by Yamaguchi et al. [9] and 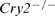 mutant neurons (compare with Figure 6E in the article by Liu et al. [10])), calculated according to Equation 9, Methods, translated into a polar plot (90 degrees corresponds to 6 hours CT). The phases exhibit a somewhat larger spread for the damped model compared with the self-sustained model, while the difference between WT and 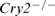 mutants is small.

We calculated the entrainment phase of each neuron, assuming a sinusoidal entrainment signal of arbitrary amplitude, using Equation 9 in Methods together with the fitted parameters. This can be done exactly for the damped model, and with the approximation outlined above for the self-sustained model. In the original study Liu et al. [10], the phases in an intact 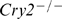 slice were given in their Figure 6E. We present the phases that the dissociated 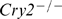 neurons would exhibit when entrained to the slice period (about 28 h), in Figure 5, upper part. One may note that the spread of phases is bigger when assuming the damped model (180 degrees, corresponding to 14 h since the period of the slice is about 28 h) than when assuming the self-sustained model (almost all phases are within a pie slice of 90 degrees, corresponding to 7 h). The entrained phase spread observed by Liu et al. [10] is smaller than 180 degrees but larger than 90 degrees. A phase spread of about 180 degrees in an intact SCN slice (WT) was found by Yamaguchi et al. [9], which can be compared to the phase spreads calculated for the WT neurons of Liu et al. [10] (Figure 5, lower part). Although all neurons of the SCN are unlikely to experience a universal synchronization signal [30], the calculations demonstrate that part of the observed phase spread can be explained by cellular heterogeneity.

**Figure 6:**
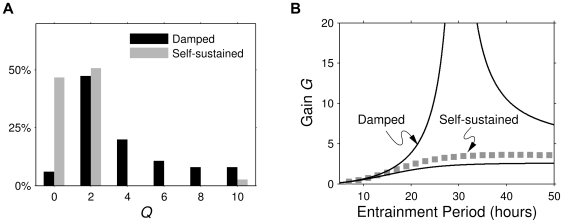
Predictions of the entrained amplitude amplifications of the cells studied. (A). Histogram of the 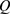 factors for the WT SCN neurons from the study of Liu et al. [10]), as calculated from the fitted parameters of the damped and self-sustained models, respectively. The 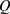 factors are a measure of the shape of the frequency response curves; the higher the value, the sharper the resonance peak is. The 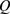 factor can be experimentally measured as the relative gain of the oscillator with respect to an entrainment signal as described in the main text. For resonant cells, i.e. cells where the frequency response curve has a maximum for a frequency other than zero, 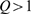. We assigned a value 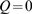 to non-resonant cells. The histogram shows that, if assuming the damped model, far fewer neurons would be non-resonant (6.0% of the neurons), than if we assume the self-sustained model (47%). Also, if assuming the damped model, we often find 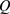 to be over 2 (58%), while this is very rare when assuming the self-sustained model (3.3%). (B). Predicted frequency response curves showing the gains 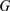, i.e. the amplitude of the driven oscillator divided by that of the forcing amplitude, for one representative 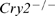 mutant SCN neuron. The damped model predicts a quite high, sharp frequency response curve (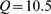), while the self-sustained model predicts a frequency response curve almost without any resonance peak (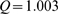). The latter frequency response curve was calculated under the approximation that the self-sustained model under forcing can be approximated as a damped oscillator with the relaxation rate to the self-sustained oscillation acting as the damping rate. The gray squares are the gains calculated from numerical simulations of the self-sustained model with a forcing term conservatively estimated from experimental data. The gains from the simulations agree reasonably well with the gains calculated from the analytical approximation. Parameter values, damped model: 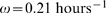, 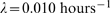. Parameter values, self-sustained model: 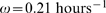, 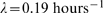, 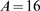. Noise intensities were set to zero.

Peripheral tissues are thought to be entrained by signals emanating from the SCN, and are known to lag behind the SCN by about 3–9 hours [31]. It follows already from our model definition that the lags must be between 0 and 12 hours (see Methods). When calculating the entrainment phase of each WT fibroblast in the Liu et al. [10] study given our two model alternatives, we obtained fairly even spreads between 0 and 12 hours (not shown). An exact prediction of the phase that would be exhibited by a population of cells is not reasonable to make, however, based on the limited study material here.

#### Predicting frequency response curves

Given the models assumed and the parameters we have fitted, frequency response curves can be calculated (as described above, and in the Methods section) along with their 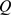 factors (Equation 2). We give in Figure 6A a histogram of the 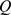 factors obtained from the 165 WT neurons in the Liu et al. [10] study. These 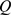 factors allow us to predict the behavior when entraining WT cells. For the cases where there is no resonance (i.e. the gain monotonically decreases with increasing frequency), we assign a 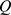 factor of zero. The histogram suggests that one may expect resonant behavior in almost all cases when assuming the damped model, and that one should observe values of 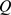 up to 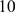. Assuming the self-sustained model, one would expect resonant behavior in only about half of the cases, and very rarely find 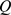 factors exceeding 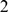. Since the 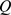 factor is the relative gain, i.e. the relative output amplitude divided by the relative input amplitude, these predictions are amenable to straightforward experimental testing.

As an example, predicted frequency response curves based on the parameters extracted from a dissociated 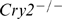 neuron, are shown in Figure 6B (solid curves). The frequency response curve for the self-sustained model was calculated assuming that it behaves as an “essentially damped” oscillator, as discussed above. To validate this approximation, we numerically simulated time courses of the entrained self-sustained model (Methods and Text S1) and calculated gains (Figure 6B, gray squares).

The striking difference between the frequency response curves estimated from the damped and the self-sustained models exemplified in Figure 6B and demonstrated statistically for all WT SCN neurons of the study in Figure 6A serves as a robust prediction that can be exploited experimentally to probe whether the circadian oscillator is damped or self-sustained at the single-cell level.

## Discussion

We have made an in-depth analysis of bioluminescence time-series data of the circadian rhythm in mouse SCN neurons and fibroblasts. The main conclusions are, first, that it is possible to estimate fundamental parameters of the oscillators, such as damping rate and noise intensity. Second, we show that the question of whether the circadian clock is self-sustained or damped is not settled, since we could reject neither hypothesis. Third, we predict frequency response curves, experimentally obtainable via e.g. temperature entrainment, that will look quite different depending on whether the rhythm is self-sustained or damped.

The parameters extracted in this study (damping rate, noise level, etc.) typically span an order of magnitude for the cell populations we study. We find it remarkable that fibroblasts and SCN neurons cluster similarly in parameter space even for the different mutants studied, which hint at a universality of the clockwork behavior. Even SCN neurons from an earlier, independent investigation [9] where a different reporter gene was used, clustered together with the WT neurons of the Liu et al. [10] study. This suggests that the parameters reflect general properties of the circadian oscillator, and that the extraction procedure we employed is robust. Another interesting property of the distributions of the estimated parameters (except for the period) is that they appear far less skewed (or even Gaussian) when viewed in logarithmic coordinates rather than linear coordinates. This is a more or less universal property of diverse biological data, such as species abundance, gene expression, and mRNA and protein copy numbers [32]–[35].

Biophysically detailed models are helpful for the conceptual understanding of how molecular properties influence the dynamics of larger integrated systems, the circadian clock being no exception [20],[21],[36]. Our modeling approach here, however, is complementary in the sense that it is top-down: it takes as starting point the dynamical features observed in single cell time-series, rather than precise knowledge of the molecular species of the system. This allows unambiguous fitting of the few parameters to data, whereas it has been shown that already moderately more complex circadian models do not allow this [37]. It is noteworthy that although the circadian clock is a very complex system, the time-series analyzed here exhibit only a few degrees of freedom and are well described by very sparse models with five or fewer parameters. On the other hand, the parameters we estimate are observational. This means that they describe the characteristics that can be observed from time-series of merely one component (PER protein), but in reality are compound parameters of a system that is much larger. A challenging problem to be solved is how the few parameters quantified here relate to kinetic and thermodynamic quantities that biophysically detailed models typically are based upon. Such quantities include transcription and translation rate constants, mRNA and protein degradation rates, protein phosphorylation rates, and equilibrium constants. It is necessary to find such relations in order to systematically observe or manipulate molecules, which will allow us to understand or alter systems level properties like circadian phase and jet-lag response, rather than relying on serendipitous discovery. On a general level, Indic et al. [38] have already shown that it indeed is possible and reasonable to reduce two different detailed biophysical circadian clock models [20],[21] to two-dimensional limit cycle models of the type considered in this study. Therefore, the results obtained in the present study should serve as constraints for biophysically detailed models. Previously, a few experimental and theoretical studies have mapped transcription rates and PER degradation rates to oscillation frequency [39]–[42].

In order for a negative feedback oscillator to be self-sustained, either strong nonlinearities or a great number of intermediate reaction steps are required. The celebrated Goodwin model [43], for instance, is never a self-sustained oscillator if the Hill coefficient is smaller than 8, independent of other parameters of the system [44]. An increased number of intermediate reaction steps lowers this Hill coefficient threshold [45]. More recently, Morelli and Jülicher [46] related the fidelity of a noisy negative feedback oscillator to the number of its constituent elementary reaction steps. In effect, the presence of noise relaxes such conditions, making the system oscillate for a broader range of parameters, i.e. also when it is damped in the absence of noise, thus enhancing robustness in this respect. Turning to our data, it is quite possible that what we observe is actually a mixture of self-sustained and damped cellular oscillators that take advantage of this principle. A consequence of this design is that noisy oscillators can have large amplitude fluctuations and peak-to-peak time variations (phase diffusion). However, it has been shown that coupling a large population of less precise SCN neurons results in a markedly more precise synchronized rhythm [7].

Virtually all theoretical descriptions of the single-cell circadian clock so far have postulated that it is a self-sustained rhythm. However, the present study suggests there is no particular reason to prefer a limit-cycle model to a damped model. Rather, neither the damped nor the self-sustained model could be rejected. Furthermore, in more than half of the WT cells, the fitted self-sustained model described an oscillation with such small amplitude in relation to the magnitude of fluctuations (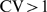 in Figure 4), that the distinction between damped and self-sustained oscillators becomes blurred. The original estimate of 66% rhythmic WT cells by Liu et al. [10] would, using our method, imply classifying cells with 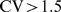 as arrhythmic, although it may be more reasonable simply to characterize cells using a continuous parameter like CV than to impose a binary classification (rhythmic or arrhythmic). In any case, a distinct difference between the predictions of the scenarios remains: the different time-scales of the amplitude relaxation rates. This is the prediction that we propose to test by measuring the frequency responses to different entrainment frequencies. In light of the fact that the observed phase spreads of intact SCN slices seems to lie in between the ones predicted by the damped model and the self-sustained model respectively, a conservative conclusion of the present study would be that the SCN is composed of a mixture of damped and self-sustained oscillators, as suggested by Aton et al. [1] in a study of the electrical activity of SCN neurons. That such a heterogeneous mixture of oscillators indeed can synchronize and entrain to an external circadian forcing has been demonstrated in a modeling study [47].

Biochemical oscillators that are damped in the deterministic sense, but driven by biochemical noise to appear self-sustained, have for a long time been hypothesized to exist [48],[49]. It is only very recently that experimental support for such ideas has begun to emerge [50]. Here, we show that time series data on the cellular circadian clock are consistent with this principle. Both the damped and the self-sustained model predict frequency response curves for the oscillation amplitude when entrained to a periodic forcing, a *zeitgeber*. Experiments should be able to settle the question as to whether these frequency response curves are actually exhibited by the circadian clock, and give further clues favoring either the damped or the self-sustained model. Such experiments will have to combine high-resolution imaging with entrainment, which can be achieved by temperature cycles [12] or light pulses [51]. We would then have a solid explanation for the phase spread of single circadian oscillators in the intact SCN [9],[10]. Together with recent advances in the theoretical understanding of the synchronization of noisy oscillators [52], one has a basis for the theoretical understanding of the SCN and the entrainment of the circadian oscillators at the organism level.

## Methods

A generic linear damped oscillator with additive white noise can be described by the following linear system of stochastic (or Langevin) differential equations:
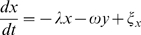


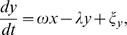
(3)where 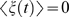, and 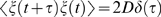. Here, the noise terms 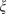 are white noise sources; 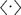 denotes time average 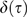 is Dirac's delta function, and the parameter 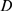 is the noise intensity. Without noise terms, this system is a damped oscillator with damping rate 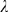 and angular frequency 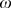. With noise terms, the system is continuously perturbed and exhibits the behavior seen in Figure 1B and E. Formally, this system could be interpreted as a negative feedback loop, where 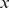 positively influences 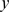, while the latter negatively influences 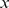. The variables 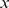 and 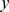 clearly must not be interpreted as absolute concentrations of chemical species, but can be interpreted as differences or distances to reference steady state concentrations. This Langevin approach belongs to the standard methods for models of stochastic gene expression, see e.g. [53]. We cannot, given our data, separate intrinsic and extrinsic noise, and hence put no specific constraints on the noise intensity 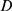, which we instead estimate from the experimental data.

Our approach to fit Equations 3 to the data is to fit the autocovariance function 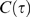, i.e. the expected values 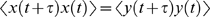, of the model to autocovariances estimated from the data. From Equations 3 one obtains (see Text S1):
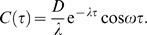
(4)


This lets us extract the damping rate 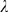, the frequency 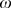, and the noise intensity 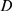 from the fit.

A generic self-sustained oscillator with linear relaxation to a limit cycle can be described in polar coordinates, i.e. radius 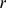 and angle 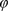, by the following system of stochastic differential equations:
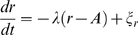


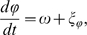
(5)where 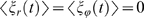, 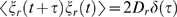, and 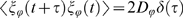. This model has two noise intensities, one for perturbations perpendicular to the limit cycle (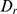), which decay with a rate 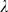, and one for perturbations along the limit cycle (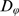). The self-sustained oscillation has an amplitude 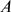, and cycles with frequency 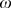. Any perturbation away from this cycle of amplitude 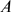 will relax back to it with a damping rate 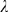. This model is a Taylor expansion to the first order around a symmetric limit cycle, and thus the parameter 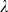 is equivalent to a Floquet exponent. The symmetry of the model is reflected by radial isochrones [54] and constant angular speed. This model class has been extensively studied by, among others, Winfree [54], also in the context of circadian rhythms. It is important to note, that without noise and with 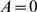, Equations 5 are just Equations 3 in radial coordinates.

Again, we calculate the autocovariance function in order to perform data fits. Transforming to Cartesian coordinates, the following autocovariance function is obtained (see Text S1):
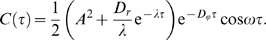
(6)


This is a cosine function multiplied by the sum of two exponential functions, which implies that in addition to a slowly relaxing term with exponent 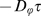, we also have a faster relaxing term with exponent 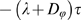.

Our approach to a distinction between self-sustained and damped oscillators rests upon the difference between one time scale of autocovariance decay, as in Equation 4, or two time scales, as in Equation 6. Crucial for this distinction is the absence of additional time scales in a damped scenario, which requires a symmetric dynamical system, as defined above. Evidence for this is the close-to-sinusoidal shape of oscillations, as validated by the good fits of the models' autocovariances to the data autocovariances. Further evidence is the fairly symmetric type 1 phase response curves [54],[55] of moderate magnitudes that are typically found in SCN neurons [56]–[60] Thus, the dynamical system is relatively symmetric, and there is probably no fixed point in the close vicinity of a limit cycle. Such a fixed point would be indicative of a highly asymmetric dynamical system (cf. e.g. [49]), which potentially could invalidate our models. Also in mammalian fibroblasts, symmetric type 1 phase response curves have been measured [51],[61],[62], although also a type 0 phase response curve was observed by Nagoshi et al. [16], perhaps reflecting the smaller absolute amplitudes of the fibroblast circadian oscillator [61]. However, this type 0 phase response curve also appeared symmetric.

When looking at entrainment properties of the cells, we consider the damped linear oscillator driven by a periodic forcing term 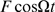, i.e.
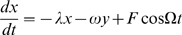


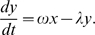
(7)


For simplicity, we here neglect noise, and we consider only the driving force acting directly on one of the variables (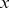). The analysis would be similar if we considered driving of both variables. A convenient framework for deriving properties of this system is provided by control theory (see Text S1 for some further details). One can thus show that the system will oscillate with the frequency 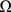 of the driving force, and that the *y*-variable of the entrained oscillator is described by the equation
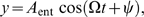
where 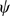 is the phase of entrainment, and 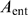 is the entrained amplitude of the variable 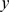. The amplitude 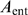 depends on the forcing frequency 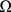 according to:
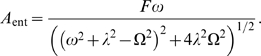
(8)


For 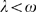, 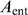 has a maximum at the resonance frequency 
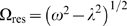
. The phase of entrainment 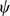 is given by:
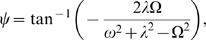
(9)where 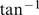 is the four-quadrant inverse tangent function, so that 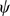 will lie in the interval 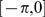. This means that the *y*-coordinate of the entrained oscillator lags behind the entrainment signal.

When studying the forced self-sustained oscillator, we apply the forcing to the Cartesian *x*-direction, in order to be able to compare to Equation 7. Thus, we consider the system
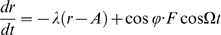


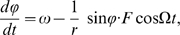
(10)when studying the entrainment of the self-sustained model.

## Supporting Information

Text S1 Supporting Information
(0.76 MB PDF)

Table S1 Statistics of data fits. For each cell type, the percentage of successful fits, as well as the fraction of cells for which the damped and self-sustained model, respectively, was rejected with *P*<0.05. The data is from the study of Liu et al. [5], except for (*), which are the neurons from the study of Yamaguchi et al. [6].
(0.02 MB PDF)

Table S2 Heterogeneity of the cell populations. Heterogeneity was quantified as the generalized standard deviation (GSD), i.e. the square root of the determinant of the covariance matrix of λ, ω, and the normalized amplitude for the damped model, and for λ, ω, the normalized amplitude, and amplitude CV (σ*_r_*/*A*) for the self-sustained model.
(0.02 MB PDF)

## References

[1] Aton SJ, Colwell CS, Harmar AJ, Waschek J, Herzog ED (2005). Vasoactive intestinal polypeptide mediates circadian rhythmicity and synchrony in mammalian clock neurons.. Nat Neurosci.

[2] Honma S, Nakamura W, Shirakawa T, Honma K (2004). Diversity in the circadian periods of single neurons of the rat suprachiasmatic nucleus depends on nuclear structure and intrinsic period.. Neurosci Lett.

[3] Liu C, Weaver DR, Strogatz SH, Reppert SM (1997). Cellular construction of a circadian clock: period determination in the suprachiasmatic nuclei.. Cell.

[4] Welsh DK, Logothetis DE, Meister M, Reppert SM (1995). Individual neurons dissociated from rat suprachiasmatic nucleus express independently phased circadian firing rhythms.. Neuron.

[5] Gonze D, Bernard S, Waltermann C, Kramer A, Herzel H (2005). Spontaneous synchronization of coupled circadian oscillators.. Biophys J.

[6] Locke JC, Westermark PO, Kramer A, Herzel H (2008). Global parameter search reveals design principles of the mammalian circadian clock.. BMC Syst Biol.

[7] Herzog ED, Aton SJ, Numano R, Sakaki Y, Tei H (2004). Temporal precision in the mammalian circadian system: a reliable clock from less reliable neurons.. J Biol Rhythms.

[8] Aton SJ, Huettner JE, Straume M, Herzog ED (2006). GABA and Gi/o differentially control circadian rhythms and synchrony in clock neurons.. Proc Natl Acad Sci U S A.

[9] Yamaguchi S, Isejima H, Matsuo T, Okura R, Yagita K (2003). Synchronization of cellular clocks in the suprachiasmatic nucleus.. Science.

[10] Liu AC, Welsh DK, Ko CH, Tran HG, Zhang EE (2007). Intercellular coupling confers robustness against mutations in the SCN circadian clock network.. Cell.

[11] Yamazaki S, Numano R, Abe M, Hida A, Takahashi R (2000). Resetting central and peripheral circadian oscillators in transgenic rats.. Science.

[12] Brown SA, Zumbrunn G, Fleury-Olela F, Preitner N, Schibler U (2002). Rhythms of mammalian body temperature can sustain peripheral circadian clocks.. Curr Biol.

[13] Kornmann B, Schaad O, Bujard H, Takahashi JS, Schibler U (2007). System-driven and oscillator-dependent circadian transcription in mice with a conditionally active liver clock.. PLoS Biol.

[14] Reddy AB, Maywood ES, Karp NA, King VM, Inoue Y (2007). Glucocorticoid signaling synchronizes the liver circadian transcriptome.. Hepatology.

[15] Balsalobre A, Damiola F, Schibler U (1998). A serum shock induces circadian gene expression in mammalian tissue culture cells.. Cell.

[16] Nagoshi E, Saini C, Bauer C, Laroche T, Naef F (2004). Circadian gene expression in individual fibroblasts: cell-autonomous and self-sustained oscillators pass time to daughter cells.. Cell.

[17] Welsh DK, Yoo SH, Liu AC, Takahashi JS, Kay SA (2004). Bioluminescence imaging of individual fibroblasts reveals persistent, independently phased circadian rhythms of clock gene expression.. Curr Biol.

[18] Hastings M, O'Neill JS, Maywood ES (2007). Circadian clocks: regulators of endocrine and metabolic rhythms.. J Endocrinol.

[19] Becker-Weimann S, Wolf J, Herzel H, Kramer A (2004). Modeling feedback loops of the mammalian circadian oscillator.. Biophys J.

[20] Forger DB, Peskin CS (2003). A detailed predictive model of the mammalian circadian clock.. Proc Natl Acad Sci U S A.

[21] Leloup JC, Goldbeter A (2003). Toward a detailed computational model for the mammalian circadian clock.. Proc Natl Acad Sci U S A.

[22] Rand DA, Shulgin BV, Salazar JD, Millar AJ (2006). Uncovering the design principles of circadian clocks: mathematical analysis of flexibility and evolutionary goals.. J Theor Biol.

[23] Raj A, van Oudenaarden A (2008). Nature, nurture, or chance: stochastic gene expression and its consequences.. Cell.

[24] Chabot JR, Pedraza JM, Luitel P, van Oudenaarden A (2007). Stochastic gene expression out-of-steady-state in the cyanobacterial circadian clock.. Nature.

[25] Mihalcescu I, Hsing W, Leibler S (2004). Resilient circadian oscillator revealed in individual cyanobacteria.. Nature.

[26] Rougemont J, Naef F (2007). Dynamical signatures of cellular fluctuations and oscillator stability in peripheral circadian clocks.. Mol Syst Biol.

[27] Brown EN, Czeisler CA (1992). The statistical analysis of circadian phase and amplitude in constant-routine core-temperature data.. J Biol Rhythms.

[28] Brown EN, Choe Y, Luithardt H, Czeisler CA (2000). A statistical model of the human core-temperature circadian rhythm.. Am J Physiol Endocrinol Metab.

[29] Hall P, Wilson SR (1991). Two guidelines for bootstrap hypothesis testing.. Biometrics.

[30] Yan L, Karatsoreos I, Lesauter J, Welsh DK, Kay S (2007). Exploring spatiotemporal organization of SCN circuits.. Cold Spring Harb Symp Quant Biol.

[31] Cermakian N, Monaco L, Pando MP, Dierich A, Sassone-Corsi P (2001). Altered behavioral rhythms and clock gene expression in mice with a targeted mutation in the Period1 gene.. EMBO J.

[32] Bengtsson M, Ståhlberg A, Rorsman P, Kubista M (2005). Gene expression profiling in single cells from the pancreatic islets of Langerhans reveals lognormal distribution of mRNA levels.. Genome Res.

[33] Hoyle DC, Rattray M, Jupp R, Brass A (2002). Making sense of microarray data distributions.. Bioinformatics.

[34] Limpert E, Stahel WA, Abbt M (2001). Log-normal distributions across the sciences: Keys and clues.. BioScience.

[35] Spencer SL, Gaudet S, Albeck JG, Burke JM, Sorger PK (2009). Non-genetic origins of cell-to-cell variability in TRAIL-induced apoptosis.. Nature.

[36] Wilkins AK, Barton PI, Tidor B (2007). The Per2 negative feedback loop sets the period in the mammalian circadian clock mechanism.. PLoS Comput Biol.

[37] Sedoglavic A (2002). A probabilistic algorithm to test local algebraic observability in polynomial time.. J Symbolic Comput.

[38] Indic P, Gurdziel K, Kronauer RE, Klerman EB (2006). Development of a two-dimension manifold to represent high dimension mathematical models of the intracellular mammalian circadian clock.. J Biol Rhythms.

[39] Dibner C, Sage D, Unser M, Bauer C, d'Eysmond T (2009). Circadian gene expression is resilient to large fluctuations in overall transcription rates.. EMBO J.

[40] Eide EJ, Woolf MF, Kang H, Woolf P, Hurst W (2005). Control of mammalian circadian rhythm by CKIepsilon-regulated proteasome-mediated PER2 degradation.. Mol Cell Biol.

[41] Reischl S, Vanselow K, Westermark PO, Thierfelder N, Maier B (2007). Beta-TrCP1-mediated degradation of PERIOD2 is essential for circadian dynamics.. J Biol Rhythms.

[42] Vanselow K, Vanselow JT, Westermark PO, Reischl S, Maier B (2006). Differential effects of PER2 phosphorylation: molecular basis for the human familial advanced sleep phase syndrome (FASPS).. Genes Dev.

[43] Goodwin BC (1965). Oscillatory behavior in enzymatic control processes.. Adv Enzyme Regul.

[44] Griffith JS (1968). Mathematics of cellular control processes. I. Negative feedback to one gene.. J Theor Biol.

[45] Rapp P (1976). Analysis of biochemical phase shift oscillators by a harmonic balancing technique.. J Math Biol.

[46] Morelli LG, Jülicher F (2007). Precision of genetic oscillators and clocks.. Phys Rev Lett.

[47] To TL, Henson MA, Herzog ED, Doyle FJ (2007). A molecular model for intercellular synchronization in the mammalian circadian clock.. Biophys J.

[48] Ebeling W, Herzel H, Richert W, Schimansky-Geier L (1986). Influence of noise on Duffing-Van der Pol oscillators.. Z angew Math Mech.

[49] Qian H, Saffarian S, Elson EL (2002). Concentration fluctuations in a mesoscopic oscillating chemical reaction system.. Proc Natl Acad Sci U S A.

[50] Skupin A, Kettenmann H, Winkler U, Wartenberg M, Sauer H (2008). How does intracellular Ca2+ oscillate: by chance or by the clock?. Biophys J.

[51] Pulivarthy SR, Tanaka N, Welsh DK, De Haro L, Verma IM (2007). Reciprocity between phase shifts and amplitude changes in the mammalian circadian clock.. Proc Natl Acad Sci U S A.

[52] Zhou T, Chen L, Aihara K (2005). Molecular communication through stochastic synchronization induced by extracellular fluctuations.. Phys Rev Lett.

[53] Ozbudak EM, Thattai M, Kurtser I, Grossman AD, van Oudenaarden A (2002). Regulation of noise in the expression of a single gene.. Nat Genet.

[54] Winfree AT (1980). The Geometry of Biological Time..

[55] Granada A, Hennig RM, Ronacher B, Kramer A, Herzel H (2009). Phase response curves elucidating the dynamics of coupled oscillators.. Methods Enzymol.

[56] Best JD, Maywood ES, Smith KL, Hastings MH (1999). Rapid resetting of the mammalian circadian clock.. J Neurosci.

[57] Gribkoff VK, Pieschl RL, Wisialowski TA, van den Pol AN, Yocca FD (1998). Phase shifting of circadian rhythms and depression of neuronal activity in the rat suprachiasmatic nucleus by neuropeptide Y: mediation by different receptor subtypes.. J Neurosci.

[58] Piggins HD, Antle MC, Rusak B (1995). Neuropeptides phase shift the mammalian circadian pacemaker.. J Neurosci.

[59] Prosser RA, Gillette MU (1989). The mammalian circadian clock in the suprachiasmatic nuclei is reset in vitro by cAMP.. J Neurosci.

[60] Wisor JP, Takahashi JS (1997). Regulation of the vgf gene in the golden hamster suprachiasmatic nucleus by light and by the circadian clock.. J Comp Neurol.

[61] Brown SA, Kunz D, Dumas A, Westermark PO, Vanselow K (2008). Molecular insights into human daily behavior.. Proc Natl Acad Sci U S A.

[62] Ukai H, Kobayashi TJ, Nagano M, Masumoto KH, Sujino M (2007). Melanopsin-dependent photo-perturbation reveals desynchronization underlying the singularity of mammalian circadian clocks.. Nat Cell Biol.

