# Supplementary material for: Quantification of Circadian Rhythms in Single Cells
Source: PLoS Comput Biol. 2009 Nov 26;5(11):e1000580. doi: 10.1371/journal.pcbi.1000580 (PMC2776301; doi:10.1371/journal.pcbi.1000580)

Text S1

Model Details

Deriving Autocovariance Functions and Stationary Probability Densities

Damped Model.

Starting from Equations 3, Methods:

$$\frac{dx}{dt} = -\lambda x - \omega y + \xi_x$$
$$\frac{dy}{dt} = \omega x - \lambda y + \xi_y, \tag{S1}$$

with $\langle \xi(t) \rangle = 0$, and $\langle \xi(t+\tau)\xi(t) \rangle = 2D\delta(\tau)$, we briefly outline how Equation 4, Methods is obtained. We denote Fourier transforms by capital letters, i.e. $\int_{-\infty}^{\infty} x(t) \, \mathrm{e}^{-i\Omega t} dt = X(\Omega)$. Fourier transforming Equations S1, solving for Y, and taking the conjugate square yields the power spectral density $YY^* = |Y|^2$:

$$|Y|^2 = \frac{2D\left(\lambda^2 + \omega^2 + \Omega^2\right)}{\left(\lambda^2 + \omega^2 - \Omega^2\right)^2 + 4\lambda^2\Omega^2}. \tag{S2}$$

The Wiener-Khinchin theorem states that the autocovariance function $C(\tau) = \langle y(t+\tau)\, y(t) \rangle$ is obtained as the Fourier transform of the power spectral density, i.e.

$$C(\tau) = \frac{1}{2\pi} \int_{-\infty}^{\infty} |Y|^2 \, \mathrm{e}^{i\Omega\tau} d\Omega.$$

Assuming $\tau \geq 0$, the classical method[1] to calculate integrals of this type is to integrate along a closed curve consisting of a line on the real axis between $-R$ and R, and a semi-circle in the complex plane stretching from R back to $-R$ via iR. Jordan's lemma dictates that as $R \to \infty$, the contribution to the curve integral from the semi-circle goes to zero since $|Y|^2$ goes to zero, and the curve integral becomes equivalent to the Fourier transform we are seeking. Jordan's lemma and the residue theorem together dictate that $\frac{1}{2\pi}\int_{-\infty}^{\infty} |Y|^2 \, \mathrm{e}^{i\Omega\tau} d\Omega = i \sum_k \mathrm{Res}\left(|Y|^2 \, \mathrm{e}^{i\Omega\tau}, \Omega_k\right)$, where $\mathrm{Res}\left(f(z), z_k\right)$ is the residue of a function $f(z)$ of a complex variable z, at the upper half-plane simple pole z_k of $f(z)$. Residues at simple poles can be calculated according to the formula $\mathrm{Res}\left(f(z), z_k\right) = \lim_{z \to z_k} (z - z_k) f(z)$. In our case, the poles are located where the denominator of the right hand side of Equation S2 vanishes, and we have two simple poles in the upper half-plane; $\Omega_1 = \omega + i\lambda$ and $\Omega_2 = -\omega + i\lambda$. With this information, it is a matter of simple algebra to derive the final result

$$C(\tau) = \frac{D}{\lambda} \mathrm{e}^{-\lambda\tau} \cos \omega\tau.$$

The stationary (time-independent, i.e. approached as $t \to \infty$) probability density $P^0(x, y)$ for the damped model is a two-dimensional Gaussian [1] which can be written in polar coordinates ($r^2 = x^2 + y^2$);

$$P^0(x, y) = \frac{\lambda}{2\pi D} \exp\left(-\frac{\lambda}{2D} r^2\right).$$

[1] The procedure is outlined in most elementary textbooks in mathematical physics.

The most probable radius (such that $\max_r P^0(r)$) is the radius yielding the maximum of the closed line integral along the angular direction: $\max_r \int_0^{2\pi} \exp\left(-\lambda r^2/(2D)\right) r d\varphi$, which is attained for $r = \sqrt{D/\lambda}$. This was used to define the amplitude of the noise-driven damped oscillator.

Self-sustained Model.

Here, we will take a different route to the autocovariance function, with an intermediate stop at the stationary probability density, which, as outlined in the main text, can be unimodal or form a crater ridge. We start from Equations 5, Methods:

$$\begin{aligned} \frac{dr}{dt} &= -\lambda (r - A) + \xi_r \\ \frac{d\varphi}{dt} &= \omega + \xi_\varphi, \end{aligned} \tag{S3}$$

with $\langle \xi_r(t) \rangle = \langle \xi_\varphi(t) \rangle = 0$, $\langle \xi_r(t+\tau)\xi_r(t) \rangle = 2D_r\delta(\tau)$ and $\langle \xi_\varphi(t+\tau)\xi_\varphi(t) \rangle = 2D_\varphi\delta(\tau)$. This particularly simple model is due to Feistel [2], but essentially the theoretical framework for noisy oscillators flows from the works of Stratonovich [3]. Note that the radial variable r is described by the Ornstein-Uhlenbeck process and is allowed to attain arbitrarily negative values (i.e. it is not the radius *per se*; this is always positive). Equations S3 are equivalent to the following Fokker-Planck equation for the probability density $P(\overline{A}, \varphi, t)$ for $\overline{A} = r - A$ and φ:

$$\frac{\partial P}{\partial t} = \frac{\partial}{\partial \overline{A}}\left(\lambda \overline{A} P + D_r \frac{\partial P}{\partial \overline{A}}\right) + \frac{\partial}{\partial \varphi}\left(-\omega P + D_\varphi \frac{\partial P}{\partial \varphi}\right). \tag{S4}$$

The solution to this Fokker-Planck equation, under the initial condition $P(\overline{A}, \varphi, 0) = \delta(\overline{A} - \overline{A}_0)\delta(\varphi - \varphi_0)$ is [1]:

$$\begin{aligned} P(\overline{A}, \varphi, t | \overline{A}_0, \varphi_0, 0) = \frac{1}{2\pi}\left(\frac{2}{\lambda} D_r D_\varphi t \left(1 - \mathrm{e}^{-2\lambda t}\right)\right)^{-1/2} \\ \times \exp\left(-\frac{\lambda}{2D_r}\frac{\left(\overline{A} - \overline{A}_0 \mathrm{e}^{-\lambda t}\right)^2}{1 - \mathrm{e}^{-2\lambda t}} - \frac{(\varphi - \omega t - \varphi_0)^2}{4D_\varphi t}\right). \end{aligned} \tag{S5}$$

As $t \to \infty$, P approaches the stationary probability density P^0, at which P no longer changes over time, i.e. $\frac{\partial P}{\partial t} = 0$. For the radial variable $\overline{A}$, this means that its stationary probability density is Gaussian:

$$P^0(\overline{A}) = \left(2\pi \frac{D_r}{\lambda}\right)^{-1/2} \exp\left(-\frac{\lambda \overline{A}^2}{2D_r}\right),$$

with a variance σ_r^2 of D_r/λ. On the other hand, the phase variable φ undergoes a random walk around an increasing mean $\omega t + \varphi_0$. Its probability density is Gaussian with variance $2D_\varphi t$, i.e. $P(\varphi, t | \varphi_0, 0) = (4\pi D_\varphi t)^{-1/2} \exp\left(-\frac{(\varphi - \omega t - \varphi_0)^2}{4D_\varphi t}\right)$. As $t \to \infty$, this density approaches a uniform density for the angular variable φ.

The stationary probability density P^0 is visualized in Figure S1. The probability density can either form a "crater ridge" (panel S1A), which represents the limit cycle oscillation, or have a single maximum, a "mountain top" (panel S1B). Which of these two possibilities is realized by P^0 depends on the coefficient of variation (CV) of the amplitude A. The amplitude and its standard deviation $\sigma_r = \sqrt{D_r/\lambda}$ (Figure S1C) together determine the CV, viz. $\mathrm{CV} = \sigma_r/A$. When $\mathrm{CV} < 1$, the probability density function forms a crater ridge, when $\mathrm{CV} \geq 1$, it forms a mountain top. This follows from elementary properties of the Gaussian probability density. The probability density along a section that cuts through the center of the limit cycle oscillation (red dashed line in Figure S1C) is the sum

of the two Gaussians centered at the respective two points where $r = A$ (panels S1D–S1F). The border between uni- and bimodality for such a sum of two Gaussians lies exactly where their standard deviations intersect (the standard deviations are marked with black dots in panels S1D–S1F).

We proceed to calculate the autocovariance function $C(\tau) = \langle y(t+\tau)\, y(t)\rangle$, where $y = (\overline{A} + A)\sin\varphi$, according to

$$
\begin{aligned}
C(\tau) &= \iint yy' P\left(y, \tau | y', 0\right) P^0\left(y'\right) dy dy' = \\
&= \lim_{t' \to \infty} \frac{1}{4\pi^2} \left(\frac{4}{\lambda^2} D_r^2 D_\varphi^2 \tau t' \left(1 - \mathrm{e}^{-2\lambda\tau}\right) \left(1 - \mathrm{e}^{-2\lambda t'}\right) \right)^{-1/2} \iiiint \left(\overline{A} + A\right) \sin\varphi \left(\overline{A}' + A\right) \sin\varphi' \\
&\quad \times \exp\left(-\frac{\lambda}{2D_r} \frac{\left(\overline{A} - \overline{A}' \mathrm{e}^{-\lambda\tau}\right)^2}{1 - \mathrm{e}^{-2\lambda\tau}} - \frac{\left(\varphi - \omega\tau - \varphi'\right)^2}{4D_\varphi \tau} \right) \\
&\quad \times \exp\left(-\frac{\lambda}{2D_r} \frac{\left(\overline{A}' - \overline{A}_0 \mathrm{e}^{-\lambda t'}\right)^2}{1 - \mathrm{e}^{-2\lambda t'}} - \frac{\left(\varphi' - \omega t' - \varphi_0\right)^2}{4D_\varphi t'} \right) d\overline{A} d\varphi d\overline{A}' d\varphi' = \\
&= \frac{1}{2} \left(A^2 + \frac{D_r}{\lambda} \mathrm{e}^{-\lambda\tau} \right) \mathrm{e}^{-D_\varphi \tau} \cos\omega\tau.
\end{aligned}
$$

Amplitude and Entrainment Phase of the Forced Oscillator

The amplitude and entrainment phase of the damped oscillator with a sinusoidal forcing term u,

$$
\begin{aligned}
\frac{dx}{dt} &= -\lambda x - \omega y + u \\
\frac{dy}{dt} &= \omega x - \lambda y,
\end{aligned}
$$

is conveniently derived within the framework of control theory [4] whereby the equation system is Fourier transformed:

$$
\begin{aligned}
X &= G\left(U - \omega Y\right) \\
Y &= FX,
\end{aligned}
$$

where $G = 1/\left(i\Omega + \lambda\right)$ and $F = \omega/\left(i\Omega + \lambda\right)$. In terms of block diagrams, this is depicted in Figure S2. Solving for Y yields $Y = UT$ with

$$
T = \frac{FG}{1 + \omega FG}.
$$

A fundamental result is that if $u = A\cos\Omega t$, then y will be a phase-shifted cosine function with a modified amplitude: $y = |T|\, A\cos\left(\Omega t + \arg T\right)$. Evaluating $|T|$ and $\arg\left(T\right)$ yields Equation 8 and 9 of the Methods section in the main text.

Simulating the Model Solutions

Solutions for the Langevin stochastic differential equations were realized numerically using the Euler-Maruyama method. Transients of 400 hours were simulated and discarded, and time-series of the same length as the experimental data were then simulated and resampled to match the sampling rates of the experimental studies (30 min for the Liu et al. [5] study and 20 min for the Yamaguchi et al. [6] study).

Approximating the Frequency Response Curve for the Self-sustained Model

The damped oscillator model without noise can be transformed to radial variables:

$$\frac{dr}{dt} = -\lambda r$$
$$\frac{d\varphi}{dt} = \omega,$$

which is equivalent to the self-sustained model with $A = 0$. When applying a forcing $F \cos \Omega t$ to the x-direction of the self-sustained model with $A > 0$ (the Methods section of the main text), forced trajectories will for sufficiently high F lie entirely outside the limit cycle, and the self-sustained model (and its frequency response curve) will behave exactly like the damped model. For smaller F, this becomes an approximation. We made a rough estimation of F from experimental data (see below) and as seen in Figure 6B of the main text, the approximation is reasonable for simulations where the estimated value of F was used.

Estimation of Model Parameters from Bioluminescence Data

Estimating the Autocovariance Function and Dealing with Measurement Noise

If we assume that measurement noise enters the bioluminescence signal y additively, we can write

$$y(t) = s(t) + r(t),$$

where $s(t)$ is the bioluminescence signal plus the mean of the measurement noise, and $r(t)$ are fluctuations of the measurement noise around the mean of the signal. We approximate these as Gaussian white noise around the noise mean, with $\langle r(t+\tau)\, r(t)\rangle = \sigma^2 \delta(k)$. Here, δ is a delta function and σ^2 is the variance of fluctuations. What we search is each estimated autocovariance C_k (with the time lag of k sampling intervals) for the biological signal;

$$C_k = \frac{1}{N-k} \sum_{i=1}^{N-k} (s_i - m_0)(s_{i+k} - m_k),$$

where N is the number of samples in the time-series, and where the means are estimated by $m_0 = \frac{1}{N-k}\sum_{i=1}^{N-k} y_i$, and $m_k = \frac{1}{N-k}\sum_{i=1}^{N-k} y_{i+k}$ (these means also include the mean of the measurement noise). Dividing the sums by $N-k$ ensures that the estimates are unbiased. The biased estimator (obtained by instead always dividing by N) is often encountered in the literature. However, it is in our case dangerous to use this, since it provides a bad estimate for short correlation times, which play a prominent part in our study.

We must however first estimate an autocovariance $\widetilde{C}_k$ that includes measurement noise, from the measured signals y_k:

$$\widetilde{C}_k = \frac{1}{N-k} \sum_{i=1}^{N-k} (y_i - m_0)(y_{i+k} - m_k),$$

It is easy to show that this estimation includes the measurement noise variance,

$$\widetilde{C}_k = C_k + \sigma^2 \delta(k).$$

What we have learned here is that the autocovariance $\widetilde{C}_k$ estimated from the measured signals contains a rapidly declining superimposed peak centered at lag zero, which stems from the measurement noise. This is, of course, under the assumption that the measurement noise has a correlation time short enough to approximate its autocorrelation as a delta function.

Fitting of Theoretical Autocovariances to Estimated Autocovariances

To each bioluminescence time series, we fit a second-degree polynomial in the least squares sense, which we then subtracted from the time series. The time series were then mean centered. After this procedure, we define them to be detrended. Autocovariances were calculated from the detrended time series as outlined above. We used only those 40 percent of the estimated autocovariances representing the shortest time lags, since, as mentioned, estimates of autocovariances for larger time lags become poor. We fit Equation 4 (Methods) to the cut estimated autocovariance functions with the ansatz

$$C(\tau) = a\mathrm{e}^{-\lambda\tau}\cos\omega\tau. \tag{S6}$$

From the fits, the three parameters D, λ, and ω are obtained, since $a = D/\lambda$. For the fits, we used a subspace trust-region optimization algorithm, as implemented in the `lsqcurvefit` subroutine of the MATLAB Statistical Toolbox (The MathWorks, Inc., V6.2 (R2008a)). In order to avoid fitting to measurement noise (see above), we fixed the autocovariance to exactly match Equation S6 for the time lag $\tau = 6T_\mathrm{s}$, where T_s is the sampling rate, for the fibroblasts of the Liu et al. [5] study. This choice was made via eyeballing of the calculated autocovariances. This yielded estimates of measurement noise standard deviations σ ranging from 0.13 to 1.2 photons per 30 minutes. To validate this, we estimated the camera noise from images taken every 30 minutes over 24 hours, in the absence of a biological sample and with settings identical to those used for the experiments reported in the Liu et al. [5] paper, and obtained a measurement noise standard deviation of 1.05 photons per 30 minutes. For the neurons of the Yamaguchi et al. [6] study, the time lag $\tau = T_\mathrm{s}$ was fixed. The zero time lag was fixed for the autocovariances of the neurons in the Liu et al. [5] study, since there were no noticeable very rapidly decreasing peaks. This is because these neurons gave rise to a very strong bioluminescence signal, drowning the measurement noise.

We found that the most robust fitting method was to fix autocovariances at the timepoints given above, and to use the optimization subroutine to fit the other two parameters (λ and ω), although fitting all three parameters globally produced very similar results. Initial guesses were obtained by first smoothing the estimated autocovariances via a low-pass filter, taking logarithms of the absolute values, then obtaining peaks via standard peak-picking. Initial guesses for damping rates were obtained by linear ordinary least-squares fitting of the logarithms of the peak magnitudes. Initial guesses for frequencies were directly obtained from the distances between the peaks.

The fit of the self-sustained model, Equation 6 (Methods) requires the ansatz

$$C(\tau) = \left(a\mathrm{e}^{-D_\varphi\tau} + b\mathrm{e}^{-\left(D_\varphi+\lambda\right)\tau}\right)\cos\omega\tau. \tag{S7}$$

After fitting, the remaining parameters of the model are retrieved as: $A = \sqrt{2a}$ and $D_r = 2\lambda b$. Furthermore, the coefficient of variation around the oscillation amplitude, $\mathrm{CV} = \sigma_r/A = \left(\sqrt{D_r/\lambda}\right)/A$, can be retrieved as $\mathrm{CV} = \sqrt{b/a}$. In contrast to the fitting of the damped model, the fitting of Equation S7 is a notoriously ill-conditioned problem [7, 8], since it involves fitting the sum of two exponentials. However, as validated below, we were able to obtain good results by obtaining initial guesses using the following classical "graphical" heuristic [8]:

1. Smooth the estimated autocovariances via a low-pass filter, take absolute values, then obtain peaks via simple peak-picking (the starting guess for the frequency is directly obtainable from the distance between the peaks).

2. Fit a straight line in the least squares sense to the logarithm of the peaks *except the first one;* this estimates the slope of the tail and hence the exponent of the exponential with slower decay, as well as a.

3. Subtract this exponential from the peaks, and fit another straight line to the logarithm of the rest. We now have the starting guess for, a, b, and the two exponents.

As for the fitting of the damped model, we then used the `lsqcurvefit` subroutine of the MATLAB Statistical Toolbox to fit the parameters, using the starting guesses obtained by the graphical method. Equation S7 was fixed to estimated autocovariances as outlined above for the damped model, reducing the number of parameters to fit to four.

Assessing the Fidelity of the Fits

We classified fits as being successful fits when meeting two criteria. First, the MATLAB `lsqcurvefit` subroutine should return an exit flag that asserts a successful fit. Second, the fitted period should be less than 50 hours. This second criterion was introduced since it makes little sense to fit an oscillator model to very slow oscillations given the length of the time-series at our disposal. We therefore excluded cells estimated to have such long "non-circadian" oscillation periods from further analysis. The percentages of successful fits for each cell type are listed in Table S1.

For each successfully fitted cell, we made 1000 simulations of the damped and self-sustained model, in each case using the fitted parameters. The simulations were made to exactly match the sampling rate and length of the experimental time-series. To the autocovariances calculated from each simulated time-series, we fit the relevant analytical autocovariance formula. The fits of the ansätze S6 and S7 to the autocovariances estimated from the simulated time-series allow us to gauge how reliable our fitting procedure is, given the sampling rate and length of the experimental data, since we in this case know the true parameter values hiding behind the simulation results. In Figure S3, the three parameters of the damped model are plotted on the abscissas, while on the ordinates, medians and error bars representing 25th and 75th percentiles of the fits to the 1000 simulations are given. For graphical reasons, we plot only 30 of the WT SCN neurons, which were chosen as to obtain a good spread in the noise intensity D. Specifically, neurons were sorted according to the estimated D value, and every third neuron was picked from the sorted series. The 30 centermost neurons in this sequence were plotted, so that a good spread was obtained, while ignoring extreme outliers. Quality of fits was thus ignored when picking the neurons. The agreement is good for all three parameters. The five parameters of the self-sustained model, together with the coefficient of variation (CV) of the amplitude (a compound parameter of the parameters λ, D_A, and A), are given in Figure S4. The WT SCN neurons were sorted according to D_A and 30 were picked for plotting independent of quality of fit, as described above. The agreement is good for all parameters, except for the phase noise intensity D_φ. This parameter represents the slowly decaying part of the autocovariance, and as mentioned, estimates of the autocovariance are poor for big time lags. However, this particular parameter plays no major role in our further analysis.

Statistical Test of the Models

The 1000 simulations and autocovariance fits made for each cell fit and model can also be exploited to test whether the respective model is reasonable at all. We here drew upon ideas outlined e.g. by Hall and Wilson [9]. The rationale is that since the simulated time series truly are generated by their respective model, the fitting to the analytical formulas for the autocovariance are as good as they can get, given the sampling rate and the length of the time series. For a model sufficiently far off the mark from the experimentally observed dynamics, the analytical autocovariance function would always be a better fit to the autocovariances of the model simulations, than to the autocovariances of the experimental data.

We reject the model if more than 95% of the 1000 simulations produced better fits than the experimental data. As a measure for the quality of fit, we used the normalized root mean square deviation (nRMSD):

$$\text{nRMSD} = \frac{\sqrt{\frac{1}{N}\sum_k \epsilon_k^2}}{\frac{1}{N}\sum_k \left|\tilde{C}_k\right|},$$

where we have an autocovariance of N time lags to fit, and where $\epsilon_k = \tilde{C}_k - C_k$ is the absolute error between the estimated and fitted analytical autocovariances at time lag τ_k. The results for each cell type are listed in Table S1.

Measuring the Heterogeneity of Cell Populations

To quantify the heterogeneity of the WT population and of each mutant population of the study of Liu et al. [5], we measured the scatter in λ, ω, and the normalized amplitude for the damped

model. For the self-sustained model, the scatter in λ, ω, the normalized amplitude, and amplitude CV (σ_r/A) were measured. Except for ω, logarithms were taken, which brought the probability densities of the observables closer to a Gaussian shape (see Figure 3 and 4 of the main text). We used the generalized standard deviation (GSD) as a measure of scatter, which is defined as the square root of the determinant of the covariance matrix of the three (damped model) and four (self-sustained model) observables, respectively. Fibroblasts and SCN neurons were lumped together, since they clustered together in parameter space. The results are summarized in Table S2.

Estimating Amplitudes and the Strength of the Entrainment Signal from Experimental Data

For Figure 3 of the main text, oscillation amplitudes for the noise-driven damped model were estimated as the most probable radius, i.e. as $\sqrt{D/\lambda}$.

For Figure 6B of the main text, we entrained the self-sustained model, and calculated gain G as the entrained amplitude A_{ent} divided by the amplitude F of the forcing signal. In order to choose a realistic forcing amplitude F, we made estimations of the differences in amplitude between dissociated $Cry2^{-/-}$ neurons and synchronized $Cry2^{-/-}$ neurons in SCN slice preparations from the Liu et al. [5] study. Amplitudes for dissociated neurons were estimated from the fits to the self-sustained model as A, giving a median value of 14 for the amplitude. Amplitudes from slice data are harder to estimate, since the neurons are relatively densely packed in intact slices. Thus, scattering from adjacent neurons contributes to the bioluminescence of a given region of interest (ROI). To estimate a background for each neuron, we defined another region just outside the ROI that did not contain any well-defined bright neurons. Then, we calculated new time series for those neurons with the background levels subtracted. Fitting the self-sustained model to this data gave an amplitude median of 46, i.e. about three times higher than for dissociated neurons. Because of this, we made the conservative estimate that the amplitudes of slice neurons are on average at least twice those of dissociated neurons. For Figure 6B of the main text, we then used the approximate frequency response curve of the self-sustained model for the chosen neuron to calculate the theoretical value of F to entrain the neuron up to the twofold higher amplitude at the oscillation period Ω_{sync} of the synchronized $Cry2^{-/-}$ slice ($\Omega_{\text{sync}} \approx 29\,\text{h}$):

$$F = 2A / \left| T\left(\Omega_{\text{sync}}\right) \right| ,$$

yielding $F \approx 14$.

References

1. Risken H (1989) The Fokker-Planck equation. Berlin: Springer, 2nd edition.

2. Feistel R (1981) Selbsterregte Schwingungen. Vortrag auf der 4. Tagung "Probleme der theoretischen Physik" vom 11.–13. Februar 1981 in Leipzig. Physikalische Gesellschaft der Deutschen Demokratischen Republik.

3. Stratonovich RL (1967) Topics in the theory of random noise, vol. 2. New York: Gordon and Breach.

4. Glad T, Ljung L (2000) Control theory: multivariable and nonlinear methods. London: Taylor & Francis.

5. Liu AC, Welsh DK, Ko CH, Tran HG, Zhang EE, et al. (2007) Intercellular coupling confers robustness against mutations in the SCN circadian clock network. Cell 129: 605–616.

6. Yamaguchi S, Isejima H, Matsuo T, Okura R, Yagita K, et al. (2003) Synchronization of cellular clocks in the suprachiasmatic nucleus. Science 302: 1408–1412.

7. Box MJ (1966) A comparison of several current optimization methods, and the use of transformations in constrained optimization. Comput J 9: 67–77.

8. Holmström K, Petersson J (2002) A review of the parameter estimation problem of fitting positive exponential sums to empirical data. Appl Math Comput 126: 31–61.

9. Hall P, Wilson SR (1991) Two guidelines for bootstrap hypothesis testing. Biometrics 47: 757–762.

Figure Captions

Figure S1

The probability density function of the self-sustained model. Whether the density function forms a "crater ridge" (panel **A**) or is unimodal (panel **B**) is determined by the coefficient of variation (CV) of the radial variable around the oscillator amplitude A (panel **C**). The CV is defined as CV $= \sigma/A$, where σ is the standard deviation σ of the radial variable around A. Along a section through the density function, illustrated by the red dashed line in panel C, the density function is the sum of two Gaussians (panel **D**). The density function is in this case bimodal, since the Gaussians overlap by less than one standard deviation (black dots), i.e. CV < 1. The rotation of this density along the angular direction gives rise to a crater ridge as in panel A. However, if CV ≥ 1, the sum of the two Gaussians is unimodal (panels **E** and **F**), and the probability density function is also unimodal, as in panel B.

Figure S2

Block diagram of the forced damped oscillator. Block diagrams of classical control theory [4] are a useful way to visualize linear dynamical systems. The variable x receives input from the forcing ($u = F \cos \Omega t$) and negative feedback ($-\omega y$) from the variable y. The variable y receives input from variable x. The blocks G and F represent the differential operators for x and y, respectively.

Figure S3

Validation of the fitting procedure. The damped oscillator. For each cell, 1000 simulations of the damped model with parameters fitted to experimental time series, were made. On the abscissas, the parameters used for the simulations are given, and on the ordinates, the parameters estimated by fits to the 1000 autocovariances estimated from the simulated time series are given (median together with upper and lower quartiles). Ideally, the estimates would all lie at the red lines of identity, but due to the finite length of the time series (same as for the experimental data), and the approximate nature of simulations and optimization algorithms, deviations occur. To avoid complete cluttering, fits for selected WT cells are shown. Selections were made automatically to achieve a good spread of the parameters in the graphs, without regard to quality of fits. Scales are log-log for D and λ, linear for ω.

Figure S4

Validation of the fitting procedure. The self-sustained oscillator. For each cell, 1000 simulations of the self-sustained model with parameters fitted to experimental time series, were made. On the abscissas, the parameters used for the simulations are given, and on the ordinates, the parameters estimated by fits to the 1000 autocovariances estimated from the simulated time series are given (median together with upper and lower quartiles). To avoid complete cluttering, fits for selected WT cells are shown. Estimates are accurate for all parameters, except D_φ, which is particularly hard to estimate, since it represents the slow time scale of Equation 6 (Methods). Autocovariance estimates for big time lags are necessarily poorer than for smaller lags. For D_φ, 17 out of 30 data points are shown. The 13 points excluded were for estimates from experimental data for which $D_\varphi < 10^{-5}$, for which, not unexpectedly, the results for the simulated data were more or less random. Finally, estimates for the CV $= \sigma/A$ are given, which is a compound parameter estimated from the ansatz Equation S7, CV $= \sqrt{b/a}$. Scales are log-log for all parameters except ω, for which the scale is linear.

Figures

Figure S1:

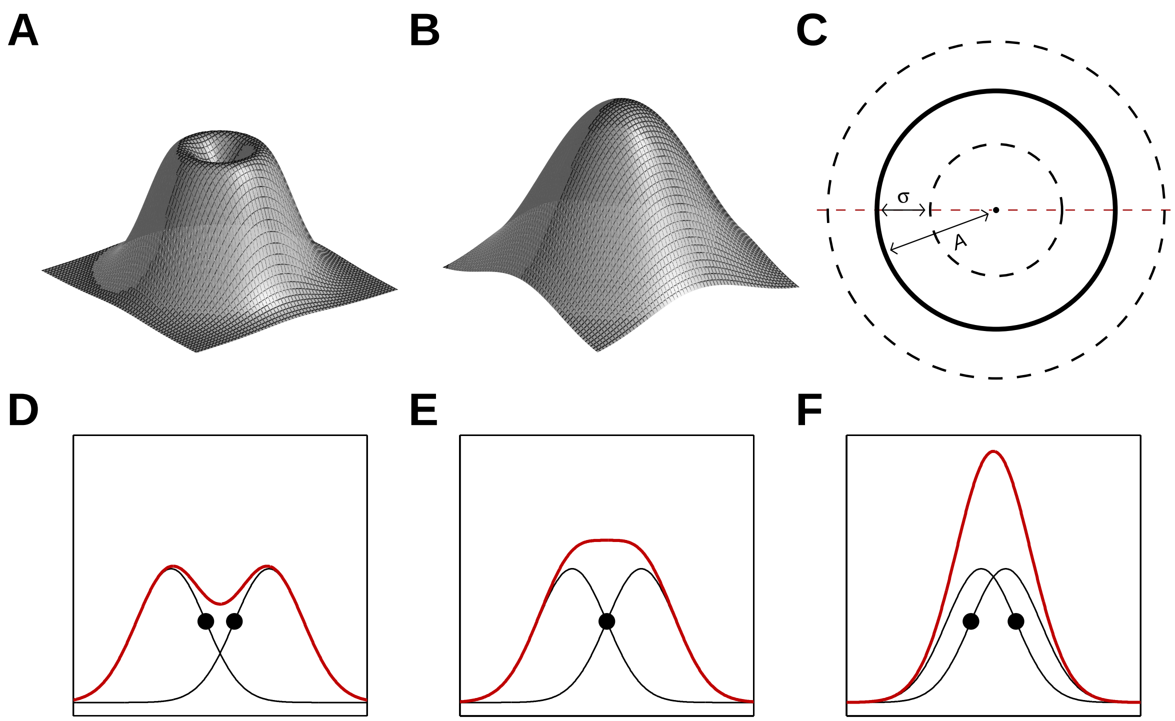

Figure S2:

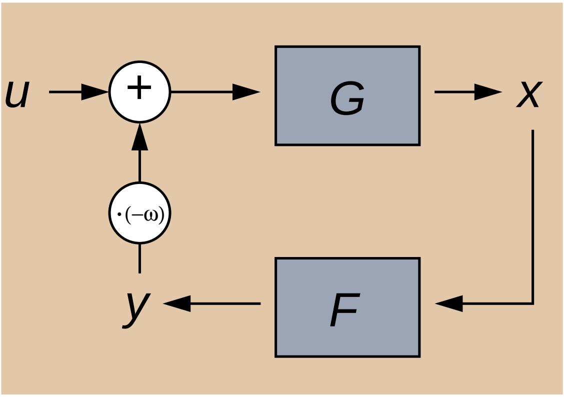

Figure S3:

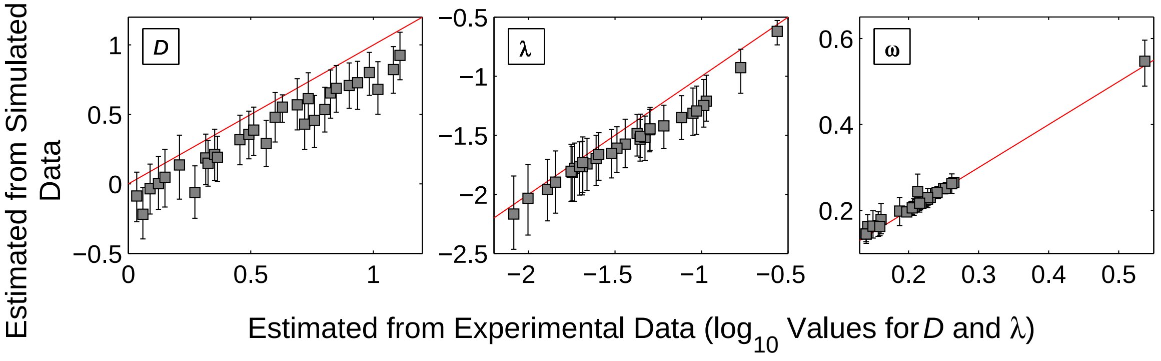

Figure S4:

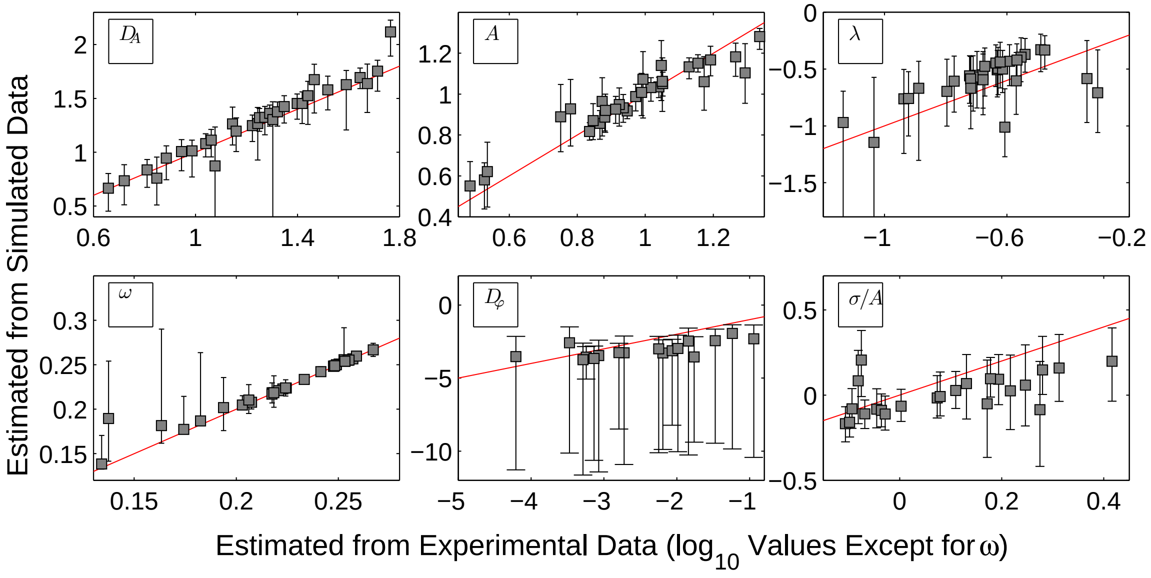

Supplement: Text S1 — Supporting Information (0.76 MB PDF) [file pcbi.1000580.s001.pdf]
