# Supplementary material for: Quantification of Circadian Rhythms in Single Cells
Source: PLoS Comput Biol. 2009 Nov 26;5(11):e1000580. doi: 10.1371/journal.pcbi.1000580 (PMC2776301; doi:10.1371/journal.pcbi.1000580)
Supplement: Table S1 — Statistics of data fits. For each cell type, the percentage of successful fits, as well as the fraction of cells for which the damped and self-sustained model, respectively, was rejected with P<0.05. The data is from the study of Liu et al. [5], except for (*), which are the neurons from the study of Yamaguchi et al. [6]. (0.02 MB PDF) [file pcbi.1000580.s002.pdf]

Table S1: **Statistics of data fits.** For each cell type, the percentage of successful fits, as well as the fraction of cells for which the damped and self-sustained model, respectively, was rejected with $P < 0.05$. The data is from the study of Liu et al. [5], except for (*), which are the neurons from the study of Yamaguchi et al. [6].

Cell type	Successful fits	Damped model rejected	Self-sustained model rejected
WT fibroblast	100%	25%	15%
Cry1$^{-/-}$ fibroblast	80%	13%	0%
Cry2$^{-/-}$ fibroblast	100%	25%	40%
Per1$^{-/-}$ fibroblast	80%	0%	13%
WT SCN neuron	88%	9.0%	2.1%
Cry1$^{-/-}$ SCN neuron	53%	4.8%	1.6%
Cry2$^{-/-}$ SCN neuron	93.2%	0.66%	7.9%
Per1$^{-/-}$ SCN neuron	60%	6.7%	2.7%
WT SCN neuron (*)	100%	1.9%	26%
