# Supplementary material for: Quantification of Circadian Rhythms in Single Cells
Source: PLoS Comput Biol. 2009 Nov 26;5(11):e1000580. doi: 10.1371/journal.pcbi.1000580 (PMC2776301; doi:10.1371/journal.pcbi.1000580)
Supplement: Table S2 — Heterogeneity of the cell populations. Heterogeneity was quantified as the generalized standard deviation (GSD), i.e. the square root of the determinant of the covariance matrix of λ, ω, and the normalized amplitude for the damped model, and for λ, ω, the normalized amplitude, and amplitude CV (σr/A) for the self-sustained model. (0.02 MB PDF) [file pcbi.1000580.s003.pdf]

Table S2: **Heterogeneity of the cell populations.** Heterogeneity was quantified as the generalized standard deviation (GSD), i.e. the square root of the determinant of the covariance matrix of λ, ω, and the normalized amplitude for the damped model, and for λ, ω, the normalized amplitude, and amplitude CV (σ_r/A) for the self-sustained model.

Cell type	GSD, damped model	GSD, self-sustained model
WT	0.029	0.019
Cry1$^{-/-}$ mutant	0.0091	0.0050
Cry2$^{-/-}$ mutant	0.014	0.00041
Per1$^{-/-}$ mutant	0.011	0.0098
